# Neonatal Maternal Separation Augments Carotid Body Response to Hypoxia in Adult Males but Not Female Rats

**DOI:** 10.3389/fphys.2016.00432

**Published:** 2016-09-27

**Authors:** Jorge Soliz, Rose Tam, Richard Kinkead

**Affiliations:** Department of Pediatrics, Centre de Recherche du CHU de Québec, Hôpital St-François d'Assise, Université LavalQuébec, QC, Canada

**Keywords:** sleep disordered breathing, hypertension, plasticity, stress

## Abstract

Perinatal exposure to adverse experiences disrupts brain development, including the brainstem network that regulates breathing. At adulthood, rats previously subjected to stress (in the form of neonatal maternal separation; NMS) display features reported in patients suffering from sleep disordered breathing, including an increased hypoxic ventilatory response and hypertension. This effect is also sex-specific (males only). Based on these observations, we hypothesized that NMS augments the carotid body's O_2_-chemosensitivity. Using an isolated and perfused *ex vivo* carotid body preparation from adult rats we compared carotid sinus nerve (CSN) responses to hypoxia and hypercapnia in carotid bodies harvested from adult rats that either experienced control conditions (no experimental manipulation) or were subjected to NMS (3 h/day from postnatal days 3 to 12). In males, the CSN response to hypoxia measured in preparations from NMS males was 1.5 fold higher than controls. In control rats, the female's response was similar to that of males; however, the increase in CSN activity measured in NMS females was 3.0 times lower than controls. The CSN response to hypercapnia was not influenced by stress or sex. We conclude that NMS is sufficient to have persistent and sex-specific effects on the carotid body's response to hypoxia. Because NMS also has sex-specific effects on the neuroendocrine response to stress, we propose that carotid body function is influenced by stress hormones. This, in turn, leads to a predisposition toward cardio-respiratory disorders.

## Introduction

Carotid bodies are small, highly vascularized chemosensors that are strategically located at the bifurcation of the carotid arteries. While they can respond to various blood born stimuli (CO_2_/H^+^, glucose), it is generally agreed that their main role is to detect changes in arterial PO_2_ reaching the brain and ultimately trigger appropriate ventilatory adjustments when necessary (Kumar and Prabhakar, 2012). Accordingly, accurate match between carotid body responsiveness to variations in arterial blood gases, ventilatory response, and metabolic demand is primordial to homeostasis; the consequences of abnormal carotid body function on cardio-respiratory health are significant.

Sleep disordered breathing affects ~4% of men and 2% for women (Jordan et al., 2014). In this population, an augmented carotid body responsiveness initiates excessive hyperventilation in response to the modest PaO_2_ fluctuations that naturally occur during sleep. The ensuing increases in PaO_2_ and CO_2_ elimination reduce respiratory drive which, in turn, exacerbate respiratory instabilities and promote apneas. Heightened hypoxic ventilatory response is therefore a hallmark of sleep disordered breathing and augmented carotid body function is key to the disease process (Younes, 2008). Because the carotid body's afferent signal also reaches pre-sympathetic neurons of the rostral ventrolateral medulla, abnormally elevated carotid body activity also contributes to cardio-vascular diseases commonly associated with sleep disordered breathing such as hypertension and congestive heart failure. Today, carotid body resection appears as a highly promising treatment of severe and resistant hypertension in humans (Iturriaga et al., 2016).

The carotid bodies and the neural pathways regulating the O_2_ chemoreflex are highly plastic. In rodents, the evidence indicating that the intermittent hypoxia resulting from repeated apneic events is sufficient to augment the O_2_ chemoreflex is compelling (Prabhakar et al., 2015). The recurrent de-oxygenation/re-oxygenation process associated with each apnea generates reactive oxygen species which then disrupt carotid body function (Prabhakar et al., 2015). Because it reproduces many of the comorbidities reported in patients, intermittent hypoxia is a valuable model to investigate the pathophysiology of sleep disordered breathing.

Chemosensory signal from the carotid bodies is also relayed to the paraventricular nucleus of the hypothalamus (Swanson and Sawchenko, 1983). For that reason, repeated carotid body activation by intermittent hypoxia can sensitize the hypothalamo-pituitary-adrenal (HPA) axis and increase circulating corticosterone (Zoccal et al., 2007; Ma et al., 2008; Coleman et al., 2010). In rats, chronic elevation of corticosterone alone is sufficient to augment the hypoxic ventilatory response (Fournier et al., 2007) and chronic associative (i.e., non-systemic) stress elicits physiological disturbances such as hypertension, inflammation, and insulin resistance commonly observed in sleep disordered breathing patients and animals subjected to intermittent hypoxia (Sparrenberger et al., 2008; Lambert and Lambert, 2011). In light of the evidence indicating that sleep disordered breathing disrupts the neuroendocrine response to stress in humans (Bratel et al., 1999; Schmoller et al., 2009), it is plausible that stress-related neuroendocrine disturbance contributes to the pathophysiology of sleep disordered breathing, including carotid body hypersensitivity.

Neonatal maternal separation (NMS) is a form of stress that interferes with programming of the HPA axis during early life (Genest et al., 2004; Buschdorf and Meaney, 2016). At adulthood, animals and humans that experienced conditions interfering with proper mother-offspring interactions (e.g., special medical care, orphanage) show an increased responsiveness to stress and are more susceptible to diseases, including hypertension and depression (Lehmann and Feldon, 2000; Marco et al., 2015). While it poses no direct stress to homeostasis, NMS also interferes with development of the cardio-respiratory network (Kinkead et al., 2013). Adult male rats previously subjected to NMS are hypertensive (20% greater) and their hypoxic ventilatory response is 25–35% higher than controls (Genest et al., 2004, 2007a). During natural sleep, respiratory instability of NMS rats is greater than controls (Kinkead et al., 2009). A remarkable feature of these effects of stress is that they are significant in males, but not females. This is important considering that the prevalence of sleep disordered breathing is ~2 times higher in men than women (Jordan et al., 2014). Together, these observations bring us to propose that neuroendocrine disruption resulting from NMS is sufficient to augment the carotid body's responsiveness to hypoxia. To address this issue, we used an *ex vivo* carotid preparation to obtain direct measurement of carotid body function. Experiments were performed on male and female rats to assess sexual dimorphism; the response to hypercapnia was also measured to evaluate stimulus-specificity of the effects.

## Materials and methods

Experiments were performed on 26 Sprague-Dawley male and female rats. Details of animal distribution amongst experimental groups and sex is provided in Table 1 and figure legends. All animals were born and raised in our animal care facilities. Dams and males used for mating were obtained from Charles River Canada (St-Constant, QC, Canada). Rats were supplied with food and water *ad libitum* and maintained in standard laboratory and animal care conditions (21°C, 12:12 dark:light cycle; lights on at 07:00 h and off at 19:00 h). Laval University Animal Care Committee approved all the experimental procedures described in this manuscript; the protocols were in accordance with the guidelines detailed by the Canadian Council on Animal Care. All experiments were performed on adult rats (see Table 1 for mean ages ± *SD*).

**Table 1:** **Comparison of mean age and weight of animals used to obtain carotid bodies for ***ex vivo*** recordings between the different experimental groups**.

	**Males**		**Females**		**Treatment effect**	**Sex effect**	**Factorial interaction**
	**Control (*n* = 8)**	**NMS (*n* = 8)**	**Control (*n* = 5)**	**NMS (*n* = 5)**			
Age (days)	66 ± 10.6	66 ± 8.6	55 ± 3.6^†^	57 ± 4.4	*P* = 0.67	***P*** = **0.007**	*P* = 0.72
Weight (g)	459 ± 54	431 ± 108	219 ± 22^†^	298 ± 51^†^	*P* = 0.41	***P*** < **0.0001**	*P* = 0.09

*Data are reported as means ± SD*.

† *Indicates a value statistically different from corresponding male value at P < 0.05. Bold indicates a significant factoral effect*.

### Mating procedures and neonatal stress protocol

Virgin females were mated and delivered 10–15 pups. Two days after delivery, litters were culled to 12 pups, when necessary, with a roughly equal number of males and females. The NMS protocol was identical to the one used in previous studies (Genest et al., 2004; Fournier et al., 2015). Briefly, the entire litter was separated from their mother for 3 h/day (09.00–12.00 h) from post-natal days 3 to 12. Separated pups were placed in a temperature- (35°C) and humidity- (45%) controlled incubator and isolated from each other by an acrylic partition. This temperature was chosen because it is within the thermoneutral range for rat pups of this age (Mortola, 2001). Data obtained from this experimental group were compared with that of animals in which the nest was not disturbed and therefore not subjected to the NMS procedure. As discussed previously (Gulemetova and Kinkead, 2011), these animals are the most desirable control group for investigations of the effects of maternal separation (Lehmann and Feldon, 2000). In each series of experiments, each group of rats was composed of animals originating from at least two litters to avoid litter-specific effects.

### *Ex vivo* electrophysiological recording of the carotid sinus nerve (CSN) activity

Rats were deeply anesthetized with ketamine/xylazine (0.1 ml/100 g, i.p.), both carotid bifurcations were removed “*en bloc*.” The excised tissue was placed in a petri dish containing ice-cold Tyrode solution (in mM: 125 NaCl, 5 KCl, 2 MgSO_4_, 1.2 NaH_2_PO_4_, 25 NaHCO_3_, 1.8 CaCl_2_, 5 sucrose, and 10 glucose, pH 7.4) bubbled with carbogen (95% O_2_ + 5% CO_2_). The carotid sinus nerve (CSN) was then carefully dissected and cleaned from surrounding connective tissue and its activity was recorded *in vitro* using standard methods used in our group (Joseph et al., 2012). Briefly, the preparation was placed in the recording chamber (volume = 5.4 ml) and a catheter placed on the inlet of the perfusion solution entering the chamber was passed through the common carotid artery to improve perfusion. The recording chamber was maintained at 36°C (TC2Bip temperature controller; Cell Micro-Controls; Norfolk, VA, USA) and perfused at a flow rate of 6 mL/min with Tyrode solution bubbled with 5% CO_2_ balance in O_2_; pH 7.4. The CSN was drawn up into the tip of a glass suction electrode (Model # 573000, A-M Systems Inc., Carlsborg, WA) for activity recording. Sufficient suction was applied to seal the electrode tip against connective tissue encircling the junction of the carotid body and CSN. A grounding electrode was placed in the recording chamber. The neural signal was fed to a differential input head-stage pre-amplifier, filtered (30–1500 Hz), and amplified (Neurolog modules NL100AK, NL104A, NL126, NL106). The signal was then processed by an A/D converter (Micro 1401-2 Cambridge Electronic Design (CED), Cambridge, UK) for display of raw activity and frequency histograms on a computer running the Spike 2 software (CED). Chemoreceptor discharges are discriminated in Spike 2 as action potentials with amplitude of 25% above baseline noise and which responded to a decrease in perfusate PO_2_ with a reversible increase in discharge frequency.

### Experimental protocol

Comparison of carotid body function between NMS and controls was assessed as follows: the experiment began when a stable CSN discharge rate was observed under baseline condition for at least 10 min (Tyrode solution bubbled with 95% O_2_ + 5% CO_2_). Then, hyperoxic recording was maintained for an additional 5 min before switching to a Tyrode solution previously equilibrated with a hypoxic gas mixture (95% N_2_ + 5% CO_2_) which was maintained for 8 min. Next, the preparation was returned to hyperoxic/normocapnic conditions (95% O_2_ + 5% CO_2_) for about 5–7 min. Once the activity had returned to basal level, recording under hypercapnic stimulation was initiated by perfusing with a Tyrode solution equilibrated with 30% CO_2_, 21% O_2_, balance N_2_. This stimulus was maintained for 8 min before returning to baseline conditions. Each carotid body preparation was used for a complete protocol.

### Data analysis and statistics

Carotid sinus nerve activity (CSN) was assessed by measuring the number of impulses above threshold per seconds on a second-by-second basis. Initially, hyperoxic activity was calculated for the baseline and recovery periods by averaging values over 150 s. The plateau of the response was obtained by averaging the activity over a 250 s period under hypoxic and hypercapnic conditions according to our previous protocol (Iturri et al., 2015). Since the hypoxic response measured in NMS females was significantly lower, these periods were extended to 200 and 300 s (baseline and hypoxic/hypercapnic stimulation, respectively) to ensure that differences were not due delays in the response. All statistical analyses were performed using Statview 5.0 (SAS Institute, Cary, NC). The effects of treatment, (control, neonatal maternal separation), and sex (male, female) and factorial interactions were assessed using a multifactorial ANOVA. The effect of stimulus (baseline, O_2_/CO_2_, recovery) was determined using a repeated measures design. The magnitude of the responses to stimuli reported in the results are expressed as a percent change from baseline. These values were used to compare responses between groups (e.g., x-fold greater than controls). Preparations that did not remain active through the entire protocol were therefore excluded from the analysis. This explains why the number of replicates is lower during in the hypercapnic test. When ANOVA revealed a significant factorial effect (or factorial interaction) *post hoc* analysis was performed using a Fisher PLSD test. All results were reported as mean ± *SD*. *P* < 0.05 was considered statistically significant.

## Results

### Comparison of carotid sinus nerve (CSN) activity under basal conditions

**Figure 1:**
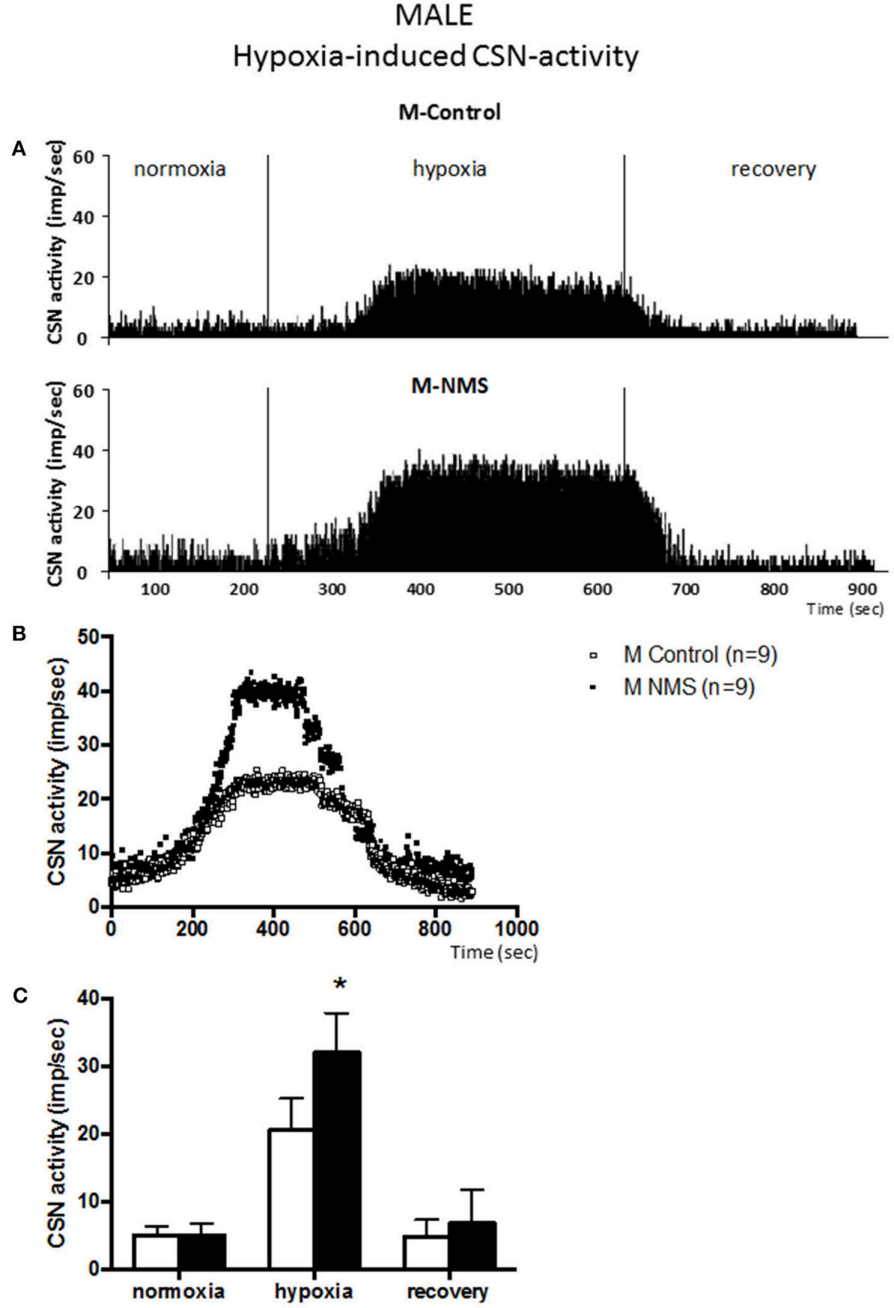
**Neonatal maternal separation (NMS) increases carotid sinus nerve (CSN) response to hypoxia in male rats. (A)** Original recordings comparing CSN activity from *ex vivo* preparations of perfused carotid body/CSN from control and NMS males under hyperoxia (FiO_2_ = 0.95), hypoxia (FiO_2_ = 0.0) and hyperoxic after hypoxia (recovery). **(B)** Comparison of the mean carotid sinus nerve activity (imp/sec) over the course of the hypoxic protocol between carotid bodies from control (open squares; *n* = 8) and NMS rats (black squares; *n* = 8). Hypoxia begins at *T* = 0 and is maintained until *T* = 500 s (end of plateau phase), followed by hyperoxic recovery; each data point represents the mean value on a second by second basis. **(C)** Histograms comparing mean CSN activity for each specific experimental condition between control (white bars; *n* = 8) and NMS (black bars; *n* = 8) male rats. Data are reported as means ± *SD*. ^*^indicates a value statistically different from control at *p* ≤ 0.05.

**Figure 2:**
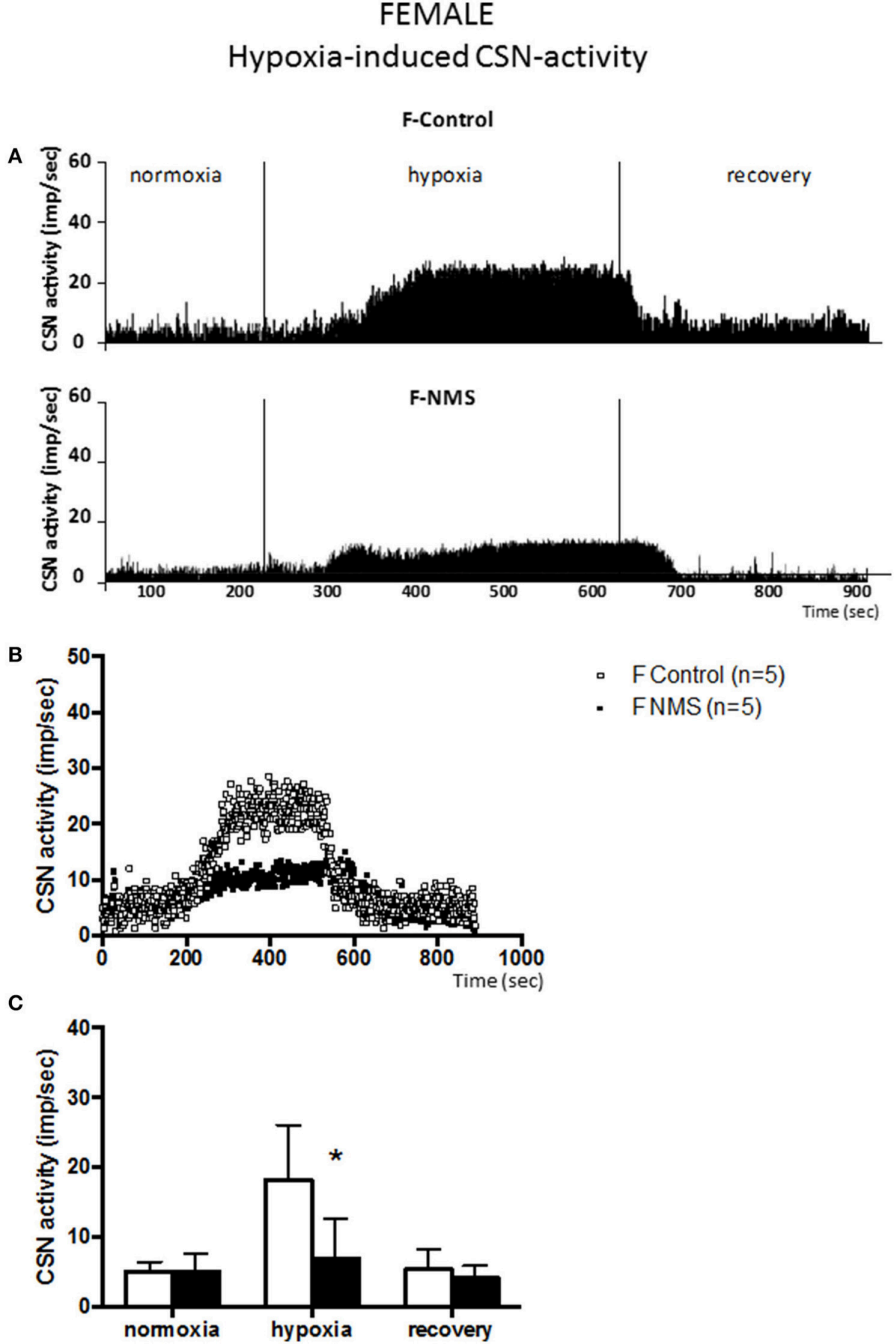
**Neonatal maternal separation (NMS) decreases carotid sinus nerve (CSN) response to hypoxia in female rats. (A)** Original recordings comparing CSN activity from *ex vivo* preparations of perfused carotid body/CSN from control and NMS females under hyperoxia (FiO_2_ = 0.95), hypoxia (FiO_2_ = 0.0) and hyperoxia after hypoxia (recovery). **(B)** Comparison of the mean carotid sinus nerve activity (imp/sec) over the course of the hypoxic protocol between carotid bodies from control (open squares; *n* = 5) and NMS rats (black squares; *n* = 5). Hypoxia begins at *T* = 0 and is maintained until *T* = 500 s (end of plateau phase), followed by hyperoxic recovery; each data point represents the mean value on a second by second basis. **(C)** Histograms comparing mean CSN activity for each specific experimental condition between control (white bars; *n* = 5) and NMS (black bars; *n* = 5) female rats. Data are reported as means ± *SD*. ^*^Indicates a value statistically different from control at *p* ≤ 0.05.

**Figure 3:**
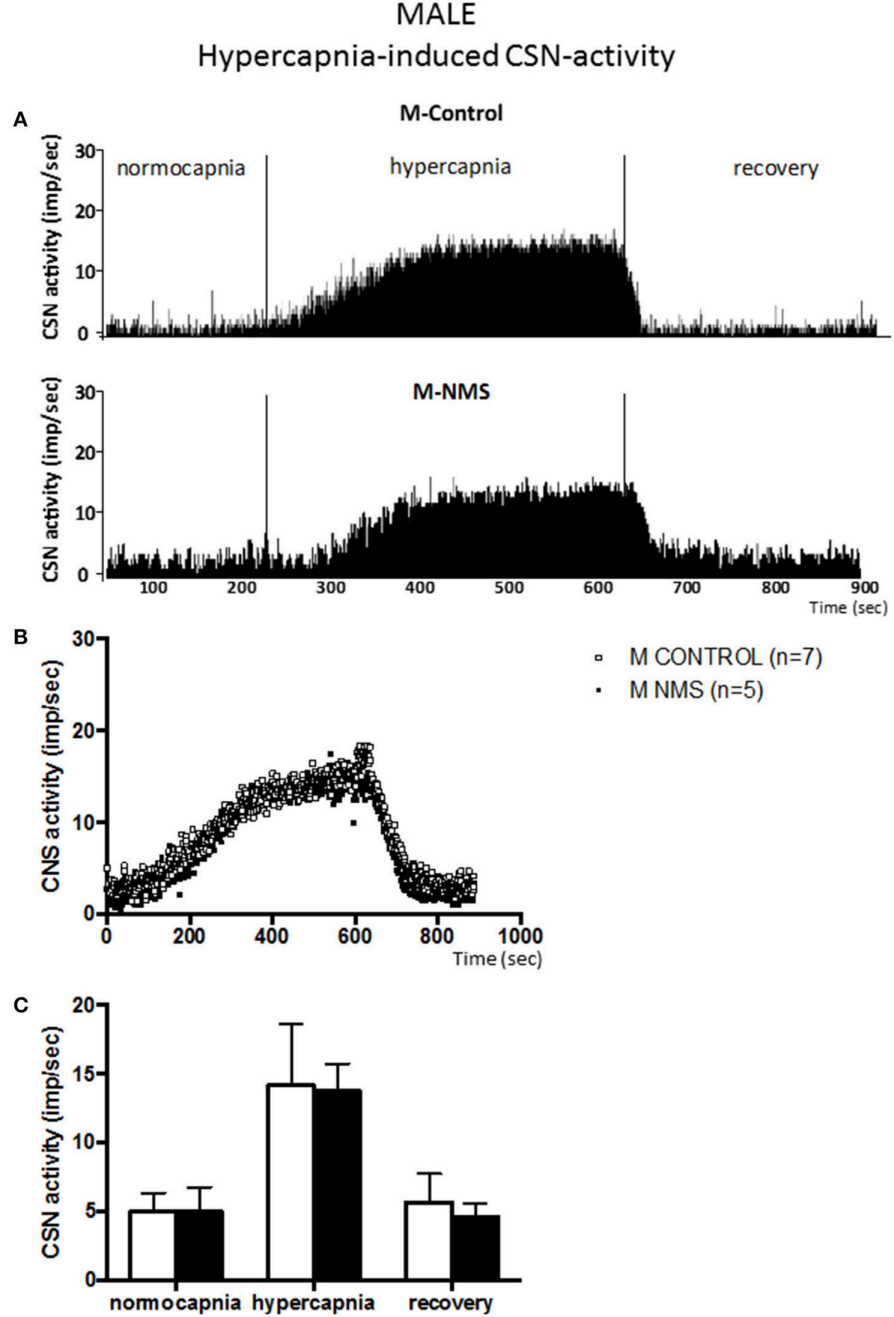
**Neonatal maternal separation (NMS) does not alter the hypercapnic carotid sinus nerve (CSN) response to hypercapnia in male rats. (A)** Original recordings comparing CSN activity from *ex vivo* preparations of perfused carotid body/CSN from control and NMS males under hyperoxia (FiO_2_ = 0.95), hypercapnia (FiCO_2_ = 0.30) and hyperoxia after hypercapnia (recovery). **(B)** Comparison of the mean carotid sinus nerve activity (imp/sec) over the course of the hypercapnic protocol between carotid bodies from control (open squares; *n* = 7) and NMS rats (black squares; *n* = 7). Hypercapnia begins at *T* = 0 and is maintained until *T* = 500 s (end of plateau phase), followed by hyperoxic recovery; each data point represents the mean value on a second by second basis. **(C)** Histograms comparing mean CSN activity for each specific experimental condition between control (white bars; *n* = 7) and NMS (black bars; *n* = 7) male rats. Data are reported as means ± *SD*.

**Figure 4:**
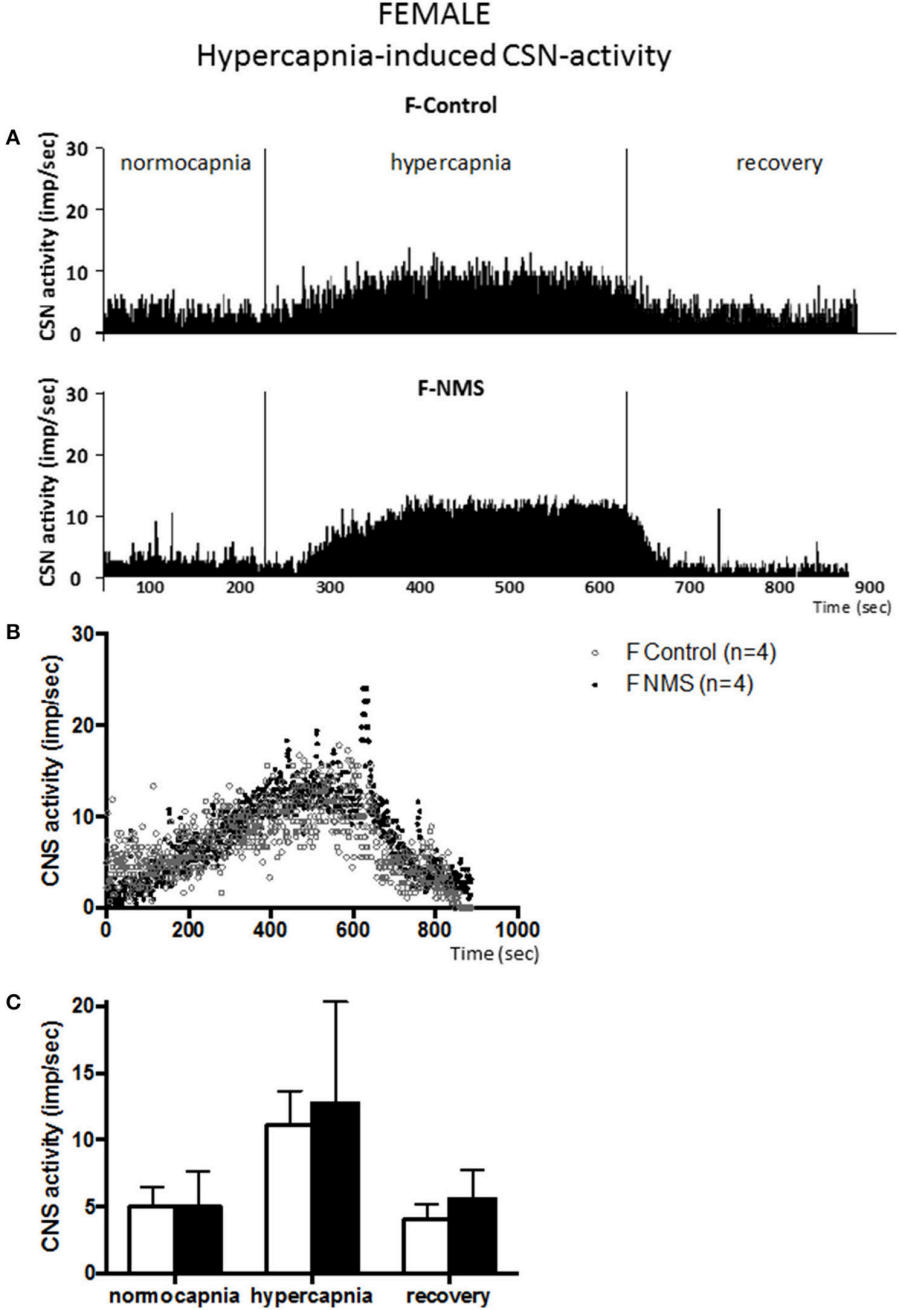
**Neonatal maternal separation (NMS) does not alter the hypercapnic carotid sinus nerve (CSN) response to hypercapnia in female rats. (A)** Original recordings comparing CSN activity from *ex vivo* preparations of perfused carotid body/CSN from control and NMS females under hyperoxia (FiO_2_ = 0.95), hypercapnia (FiCO_2_ = 0.30) and hyperoxia after hypoxia (recovery). **(B)** Comparison of the mean carotid sinus nerve activity (imp/sec) over the course of the hypercapnic protocol between carotid bodies from control (open squares; *n* = 4) and NMS rats (black squares; *n* = 4). Hypercapnia begins at *T* = 0 and is maintained until *T* = 500 s (end of plateau phase), followed by hyperoxic recovery; each data point represents the mean value on a second by second basis. **(C)** Histograms comparing mean CSN activity for each specific experimental condition between control (white bars; *n* = 4) and NMS (black bars; *n* = 4) female rats. Data are reported as means ± *SD*.

Prior to the onset of the stimulation protocol, carotid body preparations perfused with a hyperoxic solution showed discharge rates of ~5 impulses/sec (range: 1.6–7.9 impulses/sec). This basal activity did not differ between groups and was not influenced by sex (Figures 1–**4**).

### Neonatal stress augments the carotid sinus nerve (CSN) response to hypoxia in males but not females

Following baseline recording, the perfusate delivered to the chamber was changed from hyperoxia to hypoxia. Following a brief delay (~90 s), CSN activity augmented in all experimental groups [*F*_(2, 44)_ = 157.9; *p* < 0.0001; Figures 1, 2] and a plateau was observed within 3 min. During this new “steady state,” the mean CSN activity observed in NMS males was higher than controls (Figure 1). These responses represent increases of 351 ± 136% and 521 ± 151% for controls and NMS, respectively; thus, the CSN response of NMS males was 1.5-fold greater than controls. Following hypoxia, CSN activity progressively returned toward baseline levels in both groups.

In females, the dynamics of the hypoxic response were similar to those observed in males. During hypoxia, the CSN activity measured in controls was not different from males (Figure 2); the control responses represent increases in CSN activity of 351 ± 136% and 254 ± 51% for males and females, respectively. In NMS females, the intensity of the CSN response to hypoxia (84 ± 106%) was substantially less than controls (254 ± 51%). This difference represents a 3.0 fold attenuation of the response; analysis of variance confirmed that the effect of neonatal stress on the CSN response to hypoxia was sex-specific [hypoxia × treatment × sex: *F*_(2, 44)_ = 11.95; *p* < 0.0001]. Following hypoxia, the progressive recovery of the CSN activity observed in females was similar to the one observed in males.

### Hypercapnia-induced CSN-activity is not altered in male and female rats exposed to NMS

Following a delay comparable to the one reported in the previous series of experiments, changing perfusate from normo- to hypercapnic condition augmented CSN activity in all groups [*F*_(2, 30)_ = 160.45; *p* < 0.0001; Figures 3, 4]. By comparison with the hypoxic series, CSN activity ramped-up more progressively during hypercapnic exposure. The mean levels of activity reached during hypercapnia (range: 11–15 impulses/sec) were less than those achieved during hypoxia (range: 11–32 impulses/sec).

In males, the mean CSN activity measured during hypercapnia did not differ between groups (Figure 3) and the magnitude of the responses were similar (198 ± 71% and 220 ± 141% for controls and NMS, respectively). Cessation of hypercapnic stimulation resulted in a progressive decrease in CSN activity with a complete recovery by the end of the protocol.

Carotid body preparations from females responded similarly to hypercapnic stimulation. In controls, the increases in CSN activity did not differ between males and females (198 ± 71% and 151 ± 55% for males and females, respectively; Figure 3 vs. Figure 4). In NMS females, the activity recorded during hypercapnia was comparable to controls; expressing the response as a percent change from baseline confirms this result (151 ± 55% vs. 166 ± 101% for controls and NMS females, respectively); analysis of variance show that the responses did not differ between males and females [hypercapnia × sex: *F*_(2, 30)_ = 0.178; *p* = 0.84] and were not affected by neonatal stress [hypercapnia × treatment: *F*_(2, 30)_ = 1.47; *p* = 0.25]. Following hypoxia, the progressive recovery of the CSN activity observed in females was similar to the one described in males.

## Discussion

Neonatal maternal separation (NMS) is a clinically relevant model of stress with significant impact on behavior and psychological health (Gunnar, 2003; Battaglia et al., 2014). Results from animal research, combined with clinical/epidemiological studies demonstrate how exposure to such adverse conditions during early life is a powerful determinant of the developmental trajectory that can ultimately influence health outcomes (Seckl and Meaney, 1993; Battaglia et al., 1995; Heim and Nemeroff, 2001; Pryce et al., 2002; Sullivan et al., 2006). This basic neurobiological principle applies to cardio-respiratory development also. While NMS is commonly viewed as a relatively modest stressor with limited relevance to cardio-respiratory function, the evidence indicating that NMS interferes with development of this vital homeostatic system is substantial (Kinkead et al., 2013, 2014). Adult rats previously subjected to NMS display sex-specific traits reported in patients suffering from sleep disordered breathing (Dempsey et al., 2010), including an augmented hypoxic ventilatory response, hypertension, respiratory instability during natural sleep, and HPA axis dysfunction (Genest et al., 2004, 2007a; Kinkead et al., 2009). Of note, the degree of respiratory instability during sleep correlates positively with the intensity of the breathing frequency response to hypoxia (Kinkead et al., 2009). We therefore proposed that NMS-induced neuroendocrine disruption is sufficient to augment carotid body O_2_-chemosensitivity and the demonstration that NMS augments the carotid body's response to hypoxia by 1.5-fold supports this hypothesis. This result is important as it explains key aspects of the cardio-respiratory phenotype of rats subjected to NMS and provides a novel view into the origins of cardio-respiratory disorders related to carotid body dysfunction such as sleep disordered breathing.

### Critique of methods

Ovarian hormones influence the carotid body's responsiveness to hypoxia. As circulating levels fluctuate throughout the estrous cycle, the lack of knowledge of the rat's estrus stage at the time when carotid bodies were harvested is a limitation of the present work. However, hormones were washed out during the protocol such that their potential influence (at least acutely) on the measurements is reduced. Furthermore, comparison of the standard deviations values indicate that data variability is similar between sexes, thereby suggesting that the impact of the estrus cycle on our results is minimal.

Given its size and fragility, performing electrophysiological recordings on the carotid body is challenging. Several approaches have been developed, yet the *ex vivo* method is perhaps the most common since the “*en bloc*” dissection is relatively rapid and minimizes mechanical trauma to the organ. However, despite cannulation of the carotid artery and delicate removal of excessive connective tissue near the structure, gas exchange still relies on diffusion. While the use of hyperoxia is not necessary in similar preparations from newborn, pups, and juvenile rats (Niane et al., 2009; Roy et al., 2012), it is frequently used in adults (Pepper et al., 1995; Peng and Prabhakar, 2004) to facilitate diffusion and ensure viability of the preparation as the tolerance to tissue hypoxia decreases with age. Consequently, “basal” activity values reported here are hyperoxic rather than normoxic and CSN activity could differ between groups during normoxia. Although not measured here, normoxic ventilatory measurements obtained in intact rats do not support this possibility (Fournier et al., 2014, 2015).

The basal level of CSN activity we recorded (~5 impulses/sec) is slightly higher than that of a naturally perfused (*in situ*) carotid body maintained under normoxic conditions (PaO_2_ range: 90–115 mm Hg; activity range: 0.1–3 impulses/sec; single unit recording) (Vidruk et al., 2001), but compares favorably to basal (hyperoxic) values reported in other studies using similar *ex vivo* approach in a fully mature rat (Pepper et al., 1995). The carotid body's responses to hypoxia and hypercapnia have been studied extensively in male rats; however, the diversity of the approaches used (*ex vivo* vs. *in situ*), recording technique (whole nerve vs. single fiber), data normalization, developmental stage, and experimental condition (PO_2_ level at rest, intensity of the O_2_/CO_2_ stimulus) makes it difficult to make proper comparison with other studies. However, the level of activity and responses reported in males during hypoxia and hypercapnia are comparable with those reported by our group and other laboratories (Cummings and Wilson, 2005; Prabhakar et al., 2007; Iturri et al., 2015). Data from females are difficult to compare since, to the best of our knowledge, investigations of carotid body function have been performed in adult female rats.

### Neonatal stress, neuroendocrine disruption and sex-specific changes in carotid body function

For basic scientists and clinicians, deciphering the mechanisms by which perturbed mother-infant interactions compromise development and health is a fundamental issue that is not entirely resolved. Today, we know that these interactions are essential in programming the stress pathways in the offspring and that insufficient/abnormal interactions augment the neuroendocrine response to stress throughout life (Liu et al., 1997; Caldji et al., 1998; Francis and Meaney, 1999). This concept has been documented in the clinic also and much like chronic stress, enhanced responsiveness to stress is associated with elevated risk for a broad range of psychological and physiological disorders such as hypertension, asthma, states of panic and anxiety, depression, and sleep disorders (Herman and Cullinan, 1997; Dugovic et al., 1999; Rietveld et al., 1999; Lombard, 2010; McEwen and Gianaros, 2010). Interestingly, many of these disorders, including HPA axis dysfunction, are co-morbidities in sleep disordered breathing patients (Harris et al., 2009; Kumar et al., 2009; Lanfranco et al., 2010).

The persistent and sex-specific consequences of NMS on HPA axis function have been established by several laboratories, including ours (Wigger and Neumann, 1999; Genest et al., 2004). At adulthood, ACTH and corticosterone levels measured in NMS males (but not females) are higher than controls (Genest et al., 2004). Corticosterone can activate gene expression, the immune system, and promote production of reactive oxygen species, endothelin 1 and inflammatory cytokines. Since these molecules can augment carotid body function (Iturriaga et al., 2015), it is not surprising that the neuroendocrine disruption resulting from NMS augments the CSN response to hypoxia and that this effect is sex-specific. The results is consistent with observations made at the whole animal level and thus help explain the physiological phenotype of NMS rats.

By comparison with controls, mean arterial blood pressure of NMS males is augmented by 20% and their ventilatory response to moderate hypoxia (FiO_2_ = 0.12) is enhanced by 25% (Genest et al., 2004). The brisk increase in frequency response at the onset of hypoxia along with experiments performed on anesthetized rats indicate that enhancement of CB function contribute to the phenotype observed in NMS males (Kinkead et al., 2005); the 1.5 fold increase in CSN response to hypoxia supports this interpretation. Conversely, female rats exposed to NMS are not hypertensive and show a hypoxic ventilatory response which is 30% lower than controls (Genest et al., 2004). The fact that this difference was mainly due to a reduced breathing frequency response (both at the onset and late phase of hypoxia) suggested that the carotid body's O_2_-sensitivity was reduced by NMS and the results reported here support this interpretation.

Chronic elevation of circulating corticosterone plays an important role in the respiratory phenotype of NMS rats since supplementation of naïve (non-stressed) animals over a 14 day period augments the ventilatory response to moderate hypoxia (FiO_2_ = 0.12) (Fournier et al., 2007). Much like NMS, however, this effect was observed only in males even though females received the same corticosterone concentration. For reasons that are still unclear to us, this protocol failed to augment corticosterone levels in females thereby suggesting that elevated corticosterone is necessary to disrupt carotid body function.

Gonadal hormones can also contribute to the sex-specific effects of NMS on carotid body function. Much like chronic stress, NMS affects the gonadotropic axis and at adulthood, moderate hypoxia augments circulating testosterone (males) and estradiol (females) in NMS but not control rats (Fournier et al., 2011, 2014, 2015). To the best of our knowledge, the presence of androgen receptors in the carotid bodies has not been tested, yet there is strong evidence indicating that testosterone potentiates the hypoxic ventilatory response and that increased the carotid body's O_2_ responsiveness contributes to this effect (Tatsumi et al., 1994). These observations, along with the demonstration that castration attenuates the hypoxic ventilatory response in NMS rats indicate that testosterone may also contribute to sex-based differences in NMS-related increase in carotid body responsiveness to hypoxia.

Progesterone and estradiol receptors are expressed on the carotid bodies and their activation potentiates the carotid body's response to hypoxia (Hannhart et al., 1990; Joseph et al., 2013). In line with these results, reduction of ovarian hormones, either by ovariectomy or aging, attenuates the ventilatory response to hypoxia (Fournier et al., 2015). Conversely, the hypoxic response of NMS females was unchanged following hormonal decline. This reduced sensitivity to ovarian hormones likely contributes to the lower CSN response observed in NMS females; however, further studies are necessary to elucidate the underlying mechanisms.

### Stimulus specificity of the effects of neonatal stress

The carotid bodies sense O_2_ and CO_2_/H^+^ *via* distinct mechanisms (Kumar and Prabhakar, 2012) and the results reported here indicate that NMS acts specifically on factors regulating the O_2_ sensing pathway whereas CO_2_/H^+^ sensing mechanism were unaffected. This specificity is consistent time-dependent plasticity of carotid body activity that can be induced by hypoxia but not hypercapnia (Cummings and Wilson, 2005). Such difference between hypoxia and hypercapnia has been observed *in vivo* also as the effects on the hypercapnic ventilatory response which contrast significantly with the response to hypoxia. At adulthood, the hypercapnic ventilatory response of NMS females is 63% greater than controls whereas the response of NMS males is reduced by 47% (Genest et al., 2007b). Experiments in anesthetized rats (males and females) point to central mechanisms and do not indicate that the carotid bodies play a major role in these phenotypes (Dumont and Kinkead, 2010, 2011; Dumont et al., 2011); the data reported here support this interpretation.

### Perspectives and significance

The results from *ex vivo* experiments strongly suggest that neuroendocrine disruption induced by an apparently modest stress during a critical period of development is sufficient to augment the carotid body's response to hypoxia in a sex-specific manner. However, the present study did not identify which hormone(s) is/are responsible for this effect and its sex-specific manifestation. While the 1.5-fold increase in chemosensitivity may seem modest by comparison with the effects of chronic intermittent hypoxia exposure in reported in *ex vivo* studies of carotid body from adults (2.7-fold) or from pups (5.2-fold) (Pawar et al., 2008), the functional consequences are significant. The augmentation of the hypoxic ventilatory response reported in intact NMS males (25–35%) (Genest et al., 2004, 2007a) compares favorably with that reported in sleep disordered breathing patients, which is approximately 30% greater than that measured in control subjects (Narkiewicz et al., 1999). Since NMS does not appear to alter basal carotid body function, stress-related augmentation of glutamatergic transmission observed in regions regulating blood pressure (Gulemetova et al., 2013) likely explains why male NMS rats are hypertensive.

The results reported here are intriguing and clearly raise numerous questions regarding the mechanisms by which NMS exerts persistent and sex-specific augmentation of the carotid body's O_2_-chemosenstivity. However, these observations highlight the carotid body's vulnerability to neuroendocrine disruption induced by non-systemic stressors during neonatal development which, in turn, may predispose to the emergence of cardio-respiratory disorders in adults.

## Author contributions

RK: Contributed to the experimental design, data interpretation, manuscript writing and editing, and provided financial support. JS: Contributed to the experimental design, data acquisition, analysis and interpretation, manuscript writing and editing, and provided financial support. RT: Performed experiments, contributed to data acquisition and analysis and contributed to the final version of the manuscript.

### Conflict of interest statement

The authors declare that the research was conducted in the absence of any commercial or financial relationships that could be construed as a potential conflict of interest.
